# Welfare Consequences of Omitting Beak Trimming in Barn Layers

**DOI:** 10.3389/fvets.2017.00222

**Published:** 2017-12-18

**Authors:** Anja B. Riber, Lena K. Hinrichsen

**Affiliations:** ^1^Department of Animal Science, Aarhus University, Tjele, Denmark

**Keywords:** beak trimming, body wounds, injurious pecking, keel bone damage, laying hen, on-farm study, plumage damage, welfare

## Abstract

Beak trimming is used worldwide as a method of reducing the damage to feathers and skin caused by injurious pecking in laying hens. However, beak trimming also causes some welfare issues as trimming the beak results in pain and sensory loss. Due to this dilemma, there is an ongoing discussion in several European countries about whether to ban beak trimming. In this study, we investigated the welfare consequences of keeping layers with intact beaks and examined for links between injurious pecking damage and keel bone damage on an individual level. A study was conducted on 10 commercial farms housing laying hens in the barn system. Each farm participated with a flock of beak-trimmed hens (T) and a flock of non-trimmed (NT) hens that were visited around 32 and 62 weeks of age. During visits, the condition of plumage, skin, feet, and keel bone of 100 hens was assessed. Mortality was recorded by the producers. NT flocks had a lower prevalence of hens with good plumage condition around 32 weeks of age (94.1 vs. 99.6%, *P* < 0.001) and a higher prevalence of hens with poor plumage condition at 62 weeks of age (63.6 vs. 15.2%, *P* < 0.001) compared with T flocks. The prevalence of hens with keel bone deviations, with both keel bone fractures and deviations and with body wounds, was higher in NT flocks compared with T flocks at both ages (*P* < 0.001). Accumulated mortality from placement to end of production tended to be higher in NT flocks compared with T flocks (14.2 vs. 8.6%; *P* = 0.06). The prevalence of keel bone damage was higher among hens with poor plumage condition than hens with moderate/good plumage condition (31.5 vs. 22.2%; *P* < 0.001). Thus, omitting beak trimming had negative consequences for the condition of plumage, skin, and keel bone, and tended to increase mortality, highlighting the risk of reduced welfare when keeping layers with intact beaks. In addition, injurious pecking damage was found to be positively linked to keel bone damage. The causal relation is unknown, but we propose that fearfulness is an important factor.

## Introduction

Beak trimming is used worldwide as a method of reducing the damage caused by injurious pecking in laying hens. Injurious pecking, i.e., damaging pecking at feathers and skin, has been associated with poor welfare in terms of pain (1) and increased fear (2, 3). Cannibalistic pecking may also cause mortality, either directly or indirectly due to infection of the wounds (4, 5).

Although beak trimming is performed with the purpose of avoiding these welfare problems, it causes welfare issues itself. The beak of a laying hen is well innervated and contains nociceptors (sensation of pain), thermoreceptors (sensation of temperature), and mechanoreceptors (sensation of pressure and texture) (6). Beak trimming therefore results in pain and sensory loss (7, 8). As the beak is a sensitive tool used during grasping of food, preening, nest building, etc., beak trimming is considered problematic as it causes a reduction in the bird’s ability to manipulate items as observed during infestations of ectoparasites (9–11).

Due to this dilemma, there is an ongoing discussion in several European countries about whether to ban the beak trimming procedure, and in some countries (e.g., Norway, Sweden, Finland, Switzerland, and Germany), it is already banned, or a ban will be enforced in near future (e.g., Netherlands, UK). Following this trend, the trade organisation for the egg sector in Denmark, the Danish Egg Association, decided to omit beak trimming in caged layers from July 2013 and in barn and free-ranging layers from July 2014. This means that presently, all laying hens in Denmark have intact beaks, a voluntary decision made by the egg sector, as beak trimming is still permitted by law.

Few on-farm studies have been published on the welfare consequences of omitting beak trimming in laying hens. These studies focus on injurious pecking and the accompanying damage, particularly plumage damage and mortality. Most of the studies of flocks of non-trimmed hens find a negative impact on at least one of the welfare indicators investigated, e.g., increased prevalence of plumage damage (12–14), an increase in severe feather pecking rate (15), or an increase in mortality (13, 16). These on-farm studies have been carried out both in loose-housing systems (12, 14–16) and in cages (13).

Different characteristics of floor feathers, i.e., feathers dropped onto the floor either due to moulting or plucking from the birds, have previously been found to be valid welfare indicators used in relation to feather pecking. For instance, prevalence of floor feathers with pecking damage has been found to be correlated positively with feather eating and feather pecking, measured as prevalence of droppings with feather content and poor plumage condition, respectively (17). Furthermore, lower densities of floor feathers and lower proportions of short downy feathers have been found at higher occurrences of poor plumage condition (17, 18). Riber and Hinrichsen (17) found no association between poor plumage condition and prevalence of droppings containing feather content, i.e., the feathers were not eaten, and speculated whether the disappearance of feathers, especially the downy feathers, was due to higher activity or arousal levels in the flocks suffering from high levels of feather pecking.

Other welfare indicators may be affected by a change in the prevalence or impact of injurious pecking. In addition to the plumage, damaging pecking may also be directed towards the skin, including the skin of toes and combs (19, 20), and in flocks of non-trimmed hens, damage to these body parts may be more prevalent. Furthermore, an incomplete plumage, whether due to moulting or damage, has been shown to affect the flight performance negatively in different bird species (21–23). Although laying hens are not swift and elegant fliers, they use their flight feathers for balance and for controlling movements, especially in three-dimensional space (24). Severe feather pecking directed to the wings and tail, causing a poor condition of the flight feathers, may therefore generate difficulties in flight navigation. Being kept in crowded captive conditions may further increase the risk of collisions and thereby the risk of fractures, particularly keel bone fractures (25). Indeed, Donaldson et al. (26) found that keel bone damage tended to increase with declining feather coverage.

High prevalence of keel bone fractures is commonly found in commercial flocks of laying hens (27–31). Keel bone fractures may be divided into two types: (1) minor incomplete fractures, at the caudal tip of the keel bone and (2) severe fractures on the remaining part of the keel bone (32). It is commonly thought that the latter type of keel bone fractures is caused from trauma due to a fall or collision, whereas the cause of the fractures at the caudal tip is still debated.

The aims of this study were twofold. The first aim was to investigate the welfare consequences of keeping barn layers with intact beaks on commercial farms. Specifically, we investigated the effects of beak treatment (trimmed vs. non-trimmed) on the condition of plumage, skin, keel bone, and feet as well as on the floor feather characteristics in barn layers. We expected to find an increase in damage to the plumage and skin in flocks of hens with intact beaks due to the increase in damaging impact which an intact beak may have compared with a trimmed beak. This was expected to be reflected in more pecking damage to the floor feathers. Furthermore, the density of floor feathers and proportion of short (downy) feathers were expected to be lower in the non-trimmed flocks, possibly due to increased activity or arousal levels. The second aim was to determine any links between injurious pecking damage and keel bone damage on an individual level. We predicted that there would be a positive link between poor condition of the flight feathers and keel bone fractures on an individual level.

## Materials and Methods

A cross-sectional study was conducted from April 2013 to June 2016 on commercial farms housing laying hens in the barn system. The barns system in Denmark consists of either single-tiered percheries or multitiered aviaries. The participating producers were originally recruited for another research project (17, 33); 10 of the original 13 barn egg producers agreed to participate in this study. The hens were housed according to the EU regulations for barn production systems (34), i.e., the barn hens were housed at a stocking density of 9 hens/m^2^ and with no outdoor access. The flock sizes ranged between 2,000 and 13,000 (mean 7,765, median 7,000). The distribution of flocks on housing systems (single- or multitiered) and hybrids (Lohmann LSL, ISA Brown, or Lohmann Brown Lite) is presented in Table 1. Each farm participated with a flock of beak-trimmed hens (T) and a flock of non-trimmed hens [non-trimmed (NT); i.e., with intact beaks]. Each flock was visited twice with the first visit around 32 weeks of age (from here referred to as “32 weeks”; T: eight flocks at 32 weeks, one flock at 33 weeks, and one flock at 40 weeks; mean 33, median 32; NT: eight flocks at 32 weeks, one flock at 33 weeks, and one flock at 34 weeks; mean 32, median 32) and the second at 62 weeks of age (T and NT: all flocks visited at 62 weeks of age). The beak-trimmed flocks were visited from April 2013 to August 2014, and non-trimmed flocks were visited from June 2014 to June 2016. Within farm, the two flocks (T and NT) were kept under comparable conditions, e.g., similar housing system, hybrid, and management. The only exception was one farm using different hybrids in the two flocks.

**Table 1:** Number of farms according to type of housing system and hybrid.

	Single tiered	Multitiered
T, NT: Lohmann LSL	1	3
T, NT: ISA Brown	0	2
T, NT: Lohmann Brown Lite	1	2
T: ISA Brown; NT: Bovans Goldline	1	0

*T, beak-trimmed; NT, non-trimmed*.

### Data Collection and Analyses

During all visits, a total of 100 hens were caught in different areas of the house, and the condition of plumage, skin, feet, and keel bone of each bird was assessed by trained observers (33) using the scoring protocol developed in the CORE Organic project HealthyHens (35). A sample size of 100 hens was chosen based on the recommendation of the Welfare Quality^®^ protocol (36) and a sample size calculation (33).

The assessment of plumage condition was based on the method described by Tauson et al. (37), modified to include an expanded explanation for the different scores (17). A 4-point scale was used (1–4 with the highest score being the best) for five body parts (neck, back, tail, belly, and wing). The summed plumage score for the five body parts was used to determine the overall plumage condition: a score of ≤10 is regarded as a poor plumage condition, a score between 11 and 14 as moderate plumage condition, and a score ≥15 as a good plumage condition (37). Similarly, the scores of the flight feathers, i.e., tail and wings, were summed: a summed score of ≤4 is regarded as a poor flight feather condition, a score of 5 as a moderate flight feather condition, and a score ≥6 as a good flight feather condition (37). Furthermore, the tail, back, and belly were assessed for the presence of wounds by using a 4-point scale assessing the size of the wounds, but we later converted the data into whether or not the hen had one or more wounds.

Presence of both fractures (fresh and old) and deviations of keel bones were assessed by palpation. In the HealthyHens scoring protocol, the caudal tip of the keel bone is scored separately from the rest of the keel bone. Due to uncertainty at the commencement of the study about the validity of assessing fractures at the caudal tip by use of palpation, we decided not to include the lower 2 cm of the keel bone. This means that we only registered fractures that were likely to have been caused by a trauma (see Introduction). Prevalence of keel bone damage is presented using the Simplified Keel Assessment Protocol (SKAP) system (38). Finally, the feet were examined for presence of toe wounds, missing toes, and hyperkeratosis. The condition of the foot pads was scored for presence of lesions or bumble foot (dorsal swelling). The presence of the described keel bone damage and foot disorders was scored on dichotomous scales (Y/N). During the visit at 62 weeks of age, each of the assessed hens was weighed using a digital scale with increments in 20-g intervals.

In addition to the welfare assessment using animal-based indicators, the number of feathers on top of the litter at 10 randomly selected areas (each 1 m^2^) of the house was counted. The areas were randomly selected by walking 15 steps in a straight line (where possible) in different directions between areas. Following the last step, a frame (1 m × 1 m) was gently thrown, and feathers within the frame were counted. During the visit at 62 weeks of age, all feathers (T: *n* = 629 and NT: *n* = 190) from these 10 areas were collected for later examination. At the visit at 62 weeks of age, droppings (*n* = 100 per flock) were equally collected from different areas in the house. Both feathers and droppings were stored at −18°C until later examination. The feathers and droppings were examined according to the methods described by Riber and Hinrichsen (17). In short, dried droppings were cracked and visually examined for presence of feathers or pieces of feathers. The following floor feather characteristics were recorded: length of each individual feather and whether or not the feather was (a) damaged from feather pecking, (b) broken at the distal end of the central stiff shaft, i.e., the rachis, or (c) downy (>75% of the vanes along the length of the rachis being downy). Feather pecking damage differs from other types of damages to feathers. It consists of one or more large areas missing along the vane/quill of the feather, with the edge of the missing area being rough and uneven.

Mortality was recorded by the producers as part of their management practices. Seven farmers provided for both T and NT flocks a summed mortality from placement up to the end of the production period (range 70–77 weeks of age), with the exception of one farmer who culled one of the study flocks at 62 weeks of age. In addition, two farmers provided information about daily feed and water consumption as well as daily egg production, which was used to calculate average daily production/consumption per hen in weeks 32 and 62 of age using data for those 2 weeks, respectively.

### Statistical Analysis

All statistical analyses were conducted in R version 3.1.3 (39) using the R packages lme4, car, and lsmeans (40–42).

#### Effects of Beak Treatments

The effect of beak treatment was investigated on (a) clinical welfare indicators (plumage condition, keel bone fractures, keel bone deviations, skin wounds, and foot injuries) at 32 and 62 weeks, (b) the percentages of droppings with feather content at 62 weeks, (c) density of floor feathers at 62 weeks, and (d) floor feather characteristics (length and damage due to feather pecking) at 62 weeks.

The SKAP system (38) as well as overall plumage condition and flight feather condition (poor, moderate, and good) were analysed in two-way tables using a χ^2^-test for the overall effect of beak treatment (T and NT) and in proportion tests to analyse if the prevalence of the different categories differed between T and NT flocks. The body weight at 62 weeks of age was analysed using a paired *t*-test for the nine farms (excluding the farm with two different hybrids).

Data on feather length and density of floor feathers were analysed using a linear regression model, and density of floor feathers was log transformed. The remaining variables (feather pecking damage, droppings with feather content, body wounds, and foot injuries) were analysed using a logistic regression model. The regression models for the body wounds and foot injuries included age (32 and 62 weeks) of the hens and the interaction between beak treatment and age. However, the variable hyperkeratosis was analysed separately for 32 and 62 weeks of age due to converging issues of the interaction model. Broken feathers were excluded from the analyses due to uncertainty of time (before/after collection) and cause of the breakage (T: *n* = 52 and NT: *n* = 50). Data on floor feathers were missing from two farms.

All regression models contained farm as a random effect to account for the differences between farms. Within farm, the two flocks (T and NT) were kept under comparable conditions, e.g., similar housing system, hybrid and management. The only exception was one farm using different hybrids in the two flocks. However, models including and excluding this farm resulted in the same conclusions. Furthermore, we tested if models including a random effect of hybrid nested in farm explained more of the variation. The conclusion was that the model including only farm as a random effect was the most suitable model for the dataset. The effect of housing system was not tested in the models as only three farms had a single-tiered housing system. However, as the pairwise comparisons were done only for flocks from the same farm, i.e., flocks of the same hybrid reared in the same housing system, the effect of hybrid and housing system is included in the random effect of farm. All results from the regression models are presented as model probabilities (logistics) or model estimates (linear) and standard error. Results are presented as back-transformed estimates and raw SE.

Mortality was analysed using the Wilcoxon Signed Rank test with paired samples for farms reporting accumulated mortality until the end of production (>70 weeks), i.e., six farms. Data from the paired flocks, i.e., flocks originating from the same farm, were always extracted from the same age period (Start: 17 weeks of age; End: 70–77 weeks of age).

#### Links between Injurious Pecking Damage and Keel Bone Damage

Tests for correlations on an individual level between different welfare indicators (keel bone fractures, keel bone deviation, plumage damage, and body wounds) were done using a χ^2^ contingency table test of the data collected at 62 weeks of age (NT hens: *n* = 1,000; T hens: *n* = 1,000). In the tests for correlations on an individual level, the beak treatment was not taken into account.

## Results

### Effects of Beak Treatment on Different Welfare Indicators

An overall difference in plumage condition (poor, moderate, or good) was found between T and NT flocks for both ages (Table 2). At 32 weeks of age, NT flocks had a higher prevalence of hens with moderate plumage condition and a lower prevalence of hens with good plumage condition compared with T flocks. At 62 weeks of age, NT flocks had a higher prevalence of hens with poor plumage condition and a lower prevalence of hens with good plumage condition compared with T flocks. The prevalence of hens with moderate plumage condition did not differ between T and NT flocks at 62 weeks of age.

**Table 2:** Plumage condition and keel bone damage (reported using the SKAP system) in non-trimmed (NT) and T (beak-trimmed) flocks at 32 and 62 weeks of age as means.

	32 weeks			62 weeks		
	NT	T	Proportion test^f^	NT	T	Proportion test^f^
**Plumage condition^c^**						
Poor (%)	0 (0–0)	0 (0–0)	–	63.6^a^ (10–97)	15.2^b^ (0–93)	χ^2^ = 488.5
Moderate (%)	5.9^a^ (0–18)	0.4^b^ (0–4)	χ^2^ = 47.8	26.8 (3–59)	29.5 (5–54)	χ^2^ = 1.7
Good (%)	94.1^a^ (80–100)	99.6^b^ (96–100)	χ^2^ = 47.8	9.6^a^ (0–38)	55.3^b^ (2–76)	χ^2^ = 474.3
**Flight feather condition^d^**						
Poor (%)	2.8^a^ (0–14)	0.2^b^ (0–1)	χ^2^ = 21.2	66.4^a^ (25–91)	25.3^b^ (5–93)	χ^2^ = 181.6
Moderate (%)	12.1^a^ (0–34)	1.2^b^ (0–5)	χ^2^ = 94.0	19.9^a^ (1–38)	34.1^b^ (7–59)	χ^2^ = 50.4
Good (%)	85.1^a^ (61–100)	98.6^b^ (95–100)	χ^2^ = 119.9	13.7^a^ (0–45)	40.6^b^ (0–67)	χ^2^ = 338.5
**Keel bone damage^e^**						
Fracture (%)	2.4 (1–6)	3.2 (0–13)	χ^2^ = 0.9	7.1 (1–22)	6.1 (0–19)	χ^2^ = 0.7
Deviation (%)	4.1^a^ (0–11)	1.4^b^ (0–5)	χ^2^ = 12.6	14.3^a^ (0–31)	7.8^b^ (1–19)	χ^2^ = 20.8
Fracture and deviation (%)	2.1^a^ (0–8)	0.6^b^ (0–2)	χ^2^ = 7.4	12.2^a^ (2–37)	4.2^b^ (0–12)	χ^2^ = 41.5
None (%)	91.4^a^ (82–96)	94.8^b^ (86–100)	χ^2^ = 8.5	66.4^a^ (25–84)	81.9^b^ (63–99)	χ^2^ = 61.9

*Numbers in brackets indicate farm ranges*.

*P < 0.05 was used as the significance level*.

*^a,b^Values within a row within age with different superscripts differ at P < 0.001*.

*^c^Plumage condition is the summed score for the five body parts assessed: a score of ≤10 is regarded as poor, a score between 11 and 14 as moderate, and a score ≥15 as good. Chi-square test (two-way table): 32 weeks: χ^2^ = 47.8, df = 1, *P* < 0.001 and 62 weeks: χ^2^ = 620.4, df = 2, *P* < 0.001*.

*^d^Flight feather condition is the summed score of tail and wings: a score of ≤4 is regarded as poor, a score of 5 as moderate, and a score ≥6 as good. Chi-square test (two-way table): 32 weeks: χ^2^ = 121.8, df = 2, *P* < 0.001 and 62 weeks: χ^2^ = 354.8, df = 2, *P* < 0.001*.

*^e^Chi-square test (two-way table): 32 weeks: χ^2^ = 23.4, df = 3, *P* < 0.001 and 62 weeks: χ^2^ = 75.1, df = 3, *P* < 0.001*.

*^f^df = 1 in all proportion tests*.

Likewise, an overall difference in flight feather condition (poor, moderate, or good) was found between T and NT flocks for both ages (Table 2). At 32 weeks of age, NT flocks had a higher prevalence of hens with poor flight feather condition and moderate flight feather condition and a lower prevalence of hens with good flight feather condition compared with T flocks. At 62 weeks of age, NT flocks had a higher prevalence of hens with poor flight feather condition and moderate flight feather condition and a lower prevalence of hens with good flight feather condition compared with T flocks.

An overall effect of beak treatment was found at the two ages on occurrences of keel bone damage (Table 2). No differences were found between NT and T flocks, irrespective of age, in prevalence of hens having fractures. The prevalence of hens with deviations and hens with both fractures and deviations was higher in NT flocks compared with T flocks at both 32 and 62 weeks of age. The prevalence of hens having neither keel bone fractures nor deviations was lower in NT flocks compared with T flocks at both 32 and 62 weeks of age.

The results from the assessment of feathers collected on top of the litter at 62 weeks of age are shown in Table 3. The prevalence of droppings with feather content did not differ between the T and NT flocks. The prevalence of feathers with pecking damage differed between the T and NT flocks, with more feathers having feather pecking damage in NT flocks than in T flocks. The density of floor feathers was lower in NT flocks compared with T flocks. In addition, the floor feathers in the NT flocks were longer than the floor feathers in the T flocks, and the average percentage of downy feather was numerically higher in T flocks (not analysed statistically).

**Table 3:** Floor feather assessment at 62 weeks of age in non-trimmed (NT) and T (beak-trimmed) flocks (mean ± SE).

	NT	T	Statistics
Droppings with feather content (%)	5.3 ± 1.02	4.6 ± 0.92	χ^2^ = 0.34, df = 1, *P* = 0.55
Feathers with pecking damage (%)	45.3 ± 11.73^a^	28.1 ± 9.23^b^	χ^2^ = 9.45, df = 1, *P* = 0.002
Feather length (cm)	12.5 ± 0.72^a^	9.7 ± 0.70^b^	*F*_1,815_ = 63.3, *P* < 0.001
Downy (%)^c^	0.5 ± 7.3	60.6 ± 48.9	–
Density of floor feathers (feather/m^2^)^d^	3.7 ± 0.19^a^	6.9 ± 0.19^b^	*F*_1,151_ = 33.3, *P* < 0.001

*P < 0.05 was used as the significance level*.

*^a,b^Values within a row with different superscripts differ significantly*.

*^c^No statistical analysis performed as downy feathers are dependent on feather length, therefore presented as mean ± SD*.

*^d^Back-transformed estimates and model SE*.

Table 4 shows the results on prevalence of hens with body wounds and different foot injuries at 32 and 62 weeks of age in NT and T flocks. For both treatments, the prevalence of hens with body wounds increased with age, but it was higher in NT flocks at both ages. For foot-pad lesions, no difference was found between treatments within ages, but the prevalence was higher at 32 weeks of age compared with 62 weeks of age. Prevalence of bumble feet was highest in T flocks at 32 weeks of age and lowest in NT flocks at both ages. The prevalence of missing toes, toe wounds, and hyperkeratosis was low. For missing toes, the prevalence was affected by age and beak treatment with more toes missing in the T flocks and at the age of 62 weeks. The prevalence of toe wounds was affected by age and tended to be affected by beak treatment with more toe wounds in the T flocks and at the age of 32 weeks. The prevalence of hyperkeratosis did not differ between T and NT flocks at neither 32 weeks nor at 62 weeks.

**Table 4:** Prevalence (%) of hens with body wounds and different foot injuries at 32 and 62 weeks of age in non-trimmed (NT) and T (beak-trimmed) flocks.

	32 weeks		62 weeks		Statistics^e^		
	NT	T	NT	T	Age × treatment	Age	Treatment
Body wounds	16.2 ± 2.43^a^	3.5 ± 0.78^b^	27.0 ± 3.41^c^	14.1 ± 2.19^a^	χ^2^ = 15.4, *P* < 0.001		
Foot-pad lesions	9.8 ± 1.63^a^	11.7 ± 1.85^a^	5.2 ± 1.01^b^	3.7 ± 0.79^b^	χ^2^ = 4.5, *P* = 0.03		
Bumble feet	1.5 ± 0.68^ab^	4.5 ± 1.85^c^	1.9 ± 0.84^a^	0.7 ± 0.36^b^	χ^2^ = 28.4, *P* < 0.001		
Missing toes^d^	0.2 ± 0.16	0.6 ± 0.30	0.3 ± 0.18	2.0 ± 0.79		χ^2^ = 9.1, *P* = 0.003	χ^2^ = 16.6, *P* < 0.001
Toe wounds^e^	0.4 ± 0.22	1.3 ± 0.41	0.2 ± 0.14	0.3 ± 0.17		χ^2^ = 6.8, *P* = 0.009	χ^2^ = 3.8, *P* = 0.05
Hyperkeratosis	0.0 ± 0.00	0.7 ± 0.50	0.07 ± 0.08	0.3 ± 0.24	32 weeks: χ^2^ = 0.32, *P* = 0.6; 62 weeks: χ^2^ = 1.5, *P* = 0.2		

*P < 0.05 was used as the significance level*.

*^a,b,c^Significant interactions: values within a row with different superscripts differ significantly*.

*^d^Model with interaction of age and treatment did not converge, instead effect of treatment was analysed separately for 32 and 62 weeks*.

*^e^df = 1 in all tests*.

Accumulated mortality from placement to end of production differed between T and NT flocks (Wilcoxon Signed Rank test, *V* = 21, *P* = 0.03) with higher mortality in NT flocks compared with T flocks. The mean mortality in NT flocks was 18.7% (farm range: 7.2–41.0%) and in T flocks 13.6% (farm range: 4.2–38.5%). One farm experienced very high mortality in both flocks (NT flock: 41.0%; T flock: 38.5%). If this farm was excluded from the analysis, the mortality tended to be higher in NT flocks compared with T flocks (Wilcoxon Signed Rank test, *V* = 15, *P* = 0.06), with the mean mortality in NT flocks being 14.2% (farm range: 7.1–21.0%) and in T flocks 8.6% (farm range: 4.2–14.1%). At 62 weeks of age, the average body weight of the hens was higher in T flocks (1,823 g, farm range: 1,633–1,993 g) compared with NT flocks (1,757 g, farm range: 1,599–1,878 g) (*t* = 8.2, df = 899, *P* < 0.001).

Information about feed intake, water intake, and egg production at 32 and 62 weeks of age is presented in Table 5 for T and NT flocks at two farms. For both farms, a numerical increase in feed and water consumption was registered in NT flocks compared with T flocks at 62 weeks of age.

**Table 5:** Feed intake, water intake, and egg production from two farms (multitiered with brown hybrids) as well as farm specific values for the prevalence of poor plumage, keel bone fracture, and keel bone deviation.

	Farm A (ISA Brown)			Farm B (Lohmann Brown Lite)		
	Non-trimmed (NT)	T	Difference^a^ (%)	NT	T	Difference^a^
**Feed (g/hen/day)**						
32 weeks	130	131	−0.8	123	121	1.6
62 weeks	186	145	28.3	128	115	11.3
**Water (ml/hen/day)**						
32 weeks	242	229	5.7	203	213	−4.7
62 weeks	283	264	7.2	234	206	13.6
**Egg production (%)^b^**						
32 weeks	91.4	92.8	−1.5	94.6	94.2	0.4
62 weeks	–^c^	80.0	–	87.1	87.5	−0.4
**Poor plumage (%)**						
32 weeks	0	0		0	0	
62 weeks	61	17		36	0	
**Keel bone fracture (%)**						
32 weeks	4	5		14	4	
62 weeks	6	4		44	15	
**Keel bone deviation (%)**						
32 weeks	4	1		11	7	
62 weeks	20	2		68	12	

^a^Difference is calculated as: (NT − T)/T*100.

*^b^Number of egg/number of hens*.

*^c^Data on egg production was missing for week 62*.

### Links between Injurious Pecking Damage and Keel Bone Damage

The prevalence of body wounds was higher among hens with poor plumage condition compared with hens with moderate/good plumage condition (35.2 vs. 12.8%; χ^2^ = 139.7, df = 1, *P* < 0.001). Likewise, the prevalence of keel bone damage (deviation, fracture, or both) was higher among hens with poor plumage condition than hens with moderate/good plumage condition (31.5 vs. 22.2%; χ^2^ = 21.0, df = 1, *P* < 0.001). The prevalence of hens with keel bone damage (deviation, fracture, or both) was higher among hens with injurious pecking damage (poor plumage condition, body wounds, or both) compared to hens without injurious pecking damage (30.9 vs. 21.4%; χ^2^ = 22.9, df = 1, *P* < 0.001).

Looking specifically at the links between the condition of the flight feathers and the other welfare indicators, we found that the majority of hens (73.1%) having a poor flight feather condition also had a poor condition of the remaining feathered parts of the body (belly, back, and neck), whereas only 15.5% of the hens having good flight feather condition had poor condition of the remaining feathered parts of the body (χ^2^ = 670.9, df = 1, *P* < 0.001). No difference was found between hens with good condition of the flight feathers and hens with poor flight feathers in the prevalence of keel bone fractures (14.5 vs. 15.3%; χ^2^ = 0.12, df = 1, *P* = 0.725). A similar result was found with regard to keel bone deviation (18.6 vs. 20.1%; χ^2^ = 0.63, df = 1, *P* = 0.42).

## Discussion

### Effects of Beak Treatment on Different Welfare Indicators

This study showed differences between T and NT flocks in relation to a number of welfare indicators. The prevalence of poor plumage condition was higher in NT compared with T flocks, which is similar to previous findings on commercial farms (12–14). This was supported by the higher prevalence of feathers dropped on the floor (“floor feathers”) with damage resulting from feather pecking found in NT flocks compared with T flocks. By contrast, Gilani et al. (15) assessed the plumage of commercial layers during rear and at 35 weeks of age and found no effects of beak treatment (trimmed vs. non-trimmed) on the percentage of the flocks with missing feathers or when assessing the plumage of the birds individually. It is well known that the plumage condition of laying hens deteriorates with age, irrespective of beak treatment (17, 43), but it has been found that the deterioration occurs faster in flocks of non-trimmed hens compared with beak-trimmed hens [this study; (13)]. Thus, the result in the study by Gilani et al. (15) may have been different if the plumage condition had been assessed at an older age, especially since they observed a higher feather pecking rate in non-trimmed flocks. In our study, differences between beak treatments in overall plumage condition were only found at 62 weeks of age, whereas Sepeur et al. (12) found the plumage condition to be worse in non-trimmed hens already at the end of rearing compared with that of beak-trimmed hens.

Beak-trimmed hens had a higher body weight (3.6%) compared with non-trimmed hens at 62 weeks of age. Normally, the procedure of beak trimming has either a temporary negative effect or no effect on body weight gain (44–46). Therefore, the difference in body weight observed in our study seems very unlikely to be a direct effect of the procedure of beak trimming. Sandilands and Savory (44) reported no difference in body weight up to 20 weeks of age of pullets that were either beak-trimmed or non-trimmed. Neither did they find a difference in plumage condition. In this study, the increased prevalence of poor plumage condition in conjunction with the lower body weight in NT flocks could indicate that the NT hens used more energy on maintenance of the body temperature and other activities (e.g., movements away from peckers and a general increase in flightiness/anxiety due to painful injurious pecking). Leeson and Morrison (47) found a decrease in feed efficiency of 0.04 for each gram of feather lost. Likewise, Tauson and Svensson (48) reported that hens with a poor plumage condition had an energy requirement for maintenance, which was 46% higher than for hens with a good plumage condition, resulting in a 27% increase in feed intake. We only obtained data on feed consumption from two farms. However, these data did indeed show a substantial increase in feed intake in conjunction with increased plumage damage on both farms in the non-trimmed flock compared with the beak-trimmed flock at 62 weeks of age. At 32 weeks of age, where no difference in plumage condition was found between beak treatments, the feed intake was similar in T and NT flocks.

Other welfare indicators were affected by beak treatment. The prevalence of body wounds were higher in NT flocks and the mortality also tended to be higher in NT flocks, indicating more injurious pecking that either directly or indirectly may have resulted in cannibalism. A higher mortality in NT flocks has been found in previous on-farm studies of laying hens (12, 49). We also expected to find an increase in damage due to toe pecking in the NT flocks, resulting in more toe wounds and missing toes. However, this was not the case, as more toes were missing on hens in T flocks, and there tended to be more toe wounds in T flocks compared with NT flocks. It is not clear why we found the opposite of what was expected, but part of the explanation may be the low prevalence of these welfare indicators (≤2%).

### Links between Injurious Pecking Damage and Keel Bone Damage

Links were found on the individual level between injurious pecking damage and keel bone damage. For instance, a hen with a poor plumage and/or skin damage is more likely to have keel bone damage (and *vice versa*) than a hen with a good/moderate plumage condition and no skin damage. This is in accordance with the results found by Donaldson et al. (26) where keel bone damage in laying hens kept in free-range farms tended to increase with declining feather coverage. This study was not designed to determine the causal relation between injurious pecking damage and keel bone damage. However, with the resulting data, we were able to test the hypothesis that a poor condition of the flight feathers may have a negative impact on flight/navigation and therefore results in more collision/crashes in the housing system, causing an increase in keel bone damage. However, we found no support for the “poor flight feather” hypothesis, as no correlation was found between a poor condition of the flight feathers and keel bone damage. In addition, the “poor flight feather” hypothesis cannot explain an increase in keel bone deviations as a consequence of injurious pecking damage, as keel bone deviations are thought not to result from crashes, but from long-term pressure on the keel bone during roosting (50–52).

An alternative explanation to the links between injurious pecking damage and keel bone damage could be that injurious pecking in a flock may induce increased fearfulness, resulting in more movements, sudden escape behaviour, flightiness, and a general increase in arousal. In an experimental study, Lee and Craig (53) found 25-week old laying hens kept in floor pens with intact beaks to be more active than beak-trimmed hens. Also, Whay et al. (54) indeed found correlations between arousal and feather pecking and between arousal and feather damage. By using optical flow measurements to track movements within commercial flocks of layers, Lee et al. (55) showed that an important predictor of future plumage condition was disturbances, such as birds running or birds pecking at each other. Increased fearfulness may result in more uncontrolled landings and take-offs, resulting in a higher risk of crashes/collisions in the housing system and thus more keel bone fractures. Also, previous studies have shown a relation between fearfulness and availability of perches (56, 57). Keeling (58) showed that perches give birds a feeling of security reflected as a later withdrawal from an approaching stuffed predator found by birds on high perches (70 cm above ground) compared with birds with a wooden bar placed directly on the floor. We therefore speculate whether fearful birds may spent more time perching, increasing the risk of keel bone deviations.

We have no direct observations of either individual level or flock level of fearfulness, which is necessary to test this hypothesis. However, results on body weight (discussed earlier) and floor feathers indicate increased movements or sudden escape behaviour/flightiness in the non-trimmed flocks where high levels of plumage damage were found. The density of floor feathers was lower in NT flocks, but the feathers were longer and had more damage compared with T flocks. As the hens in the NT flocks did not ingest the feathers, assessed from the finding of a similar prevalence of droppings with feather content in the two beak treatments, but still had a considerable higher prevalence of poor plumage condition, feathers must have disappeared somehow else from the floor. The prevalence of downy floor feathers was much lower in NT flocks compared with T flocks, indicating that the feathers disappearing were the short light downy ones. As proposed previously (17), this could be due to increased movements or flightiness in flocks of NT hens, leading to the downy feathers disappearing into the litter or out in the corners of the building.

The direction of the relation between injurious pecking damage and keel bone damage is unknown. Above, we have argued how a poor plumage condition indirectly may result in keel bone damage. However, the opposite scenario is also possible. A hen with a good plumage condition who fractures her keel bone may show a change in behaviour, e.g., in terms of increased inactivity (59, 60). A previous study has shown that the target of severe feather pecking is most likely an inactive bird (61).

### Conclusion

It is well known that injurious pecking results in reduced welfare in terms of pain and fear due to the tearing of feathers and tissue (1–3). In addition to that, this study showed that injurious pecking is also positively linked to keel bone damage. With the move in Europe towards keeping laying hens with intact beaks, this study highlights the risk of reduced welfare of layers by omitting beak trimming. To avoid that, knowledge on prevention of injurious pecking and on action plans if outbreaks occur is essential for farmers in the daily practice. The causal relation between injurious pecking damage and keel bone damage is unknown. We propose that more injurious pecking or a greater impact of injurious pecking (as expected in hens with intact beaks) in a flock increases the level of fearfulness. Increased fearfulness may result in more keel bone fractures due to a higher risk of uncontrolled landings and take-offs and in more keel bone deviations as fearful birds may spend more time perching. Future studies should investigate this hypothesis.

## Ethics Statement

All procedures involving animals were approved by the Danish Animal Experiments Inspectorate in accordance with the Danish Ministry of Justice Law no. 382 (June 10, 1987) and Acts 333 (May 19, 1990), 726 (September 9, 1993), and 1016 (December 12, 2001).

## Author Contributions

AR conceived the idea of the project, raised funding, and collected the data. LH did the statistical analyses. The manuscript was prepared, edited, and approved by both authors.

## Conflict of Interest Statement

The authors declare that the work was conducted in the absence of any commercial or financial relationships that could be construed as a potential conflict of interest.
